# The effects of marine eukaryote evolution on phosphorus, carbon and oxygen cycling across the Proterozoic–Phanerozoic transition

**DOI:** 10.1042/ETLS20170156

**Published:** 2018-06-29

**Authors:** Timothy M. Lenton, Stuart J. Daines

**Affiliations:** Earth System Science Group, College of Life and Environmental Sciences, University of Exeter, Exeter, U.K.

**Keywords:** biogeochemistry, carbon, eukaryote, evolution, oxygen, phosphorus

## Abstract

A ‘Neoproterozoic oxygenation event’ is widely invoked as a causal factor in animal evolution, and often attributed to abiotic causes such as post-glacial pulses of phosphorus weathering. However, recent evidence suggests a series of transient ocean oxygenation events ∼660–520 Ma, which do not fit the simple model of a monotonic rise in atmospheric oxygen (pO_2_). Hence, we consider mechanisms by which the evolution of marine eukaryotes, coupled with biogeochemical and ecological feedbacks, potentially between alternate stable states, could have caused changes in ocean carbon cycling and redox state, phosphorus cycling and atmospheric pO_2_. We argue that the late Tonian ocean ∼750 Ma was dominated by rapid microbial cycling of dissolved organic matter (DOM) with elevated nutrient (P) levels due to inefficient removal of organic matter to sediments. We suggest the abrupt onset of the eukaryotic algal biomarker record ∼660–640 Ma was linked to an escalation of protozoan predation, which created a ‘biological pump’ of sinking particulate organic matter (POM). The resultant transfer of organic carbon (*C*_org_) and phosphorus to sediments was strengthened by subsequent eukaryotic innovations, including the advent of sessile benthic animals and mobile burrowing animals. Thus, each phase of eukaryote evolution tended to lower P levels and oxygenate the ocean on ∼10^4^ year timescales, but by decreasing *C*_org_/P burial ratios, tended to lower atmospheric pO_2_ and deoxygenate the ocean again on ∼10^6^ year timescales. This can help explain the transient nature and ∼10^6^ year duration of oceanic oxygenation events through the Cryogenian–Ediacaran–Cambrian.

## Introduction

**Figure 1.**
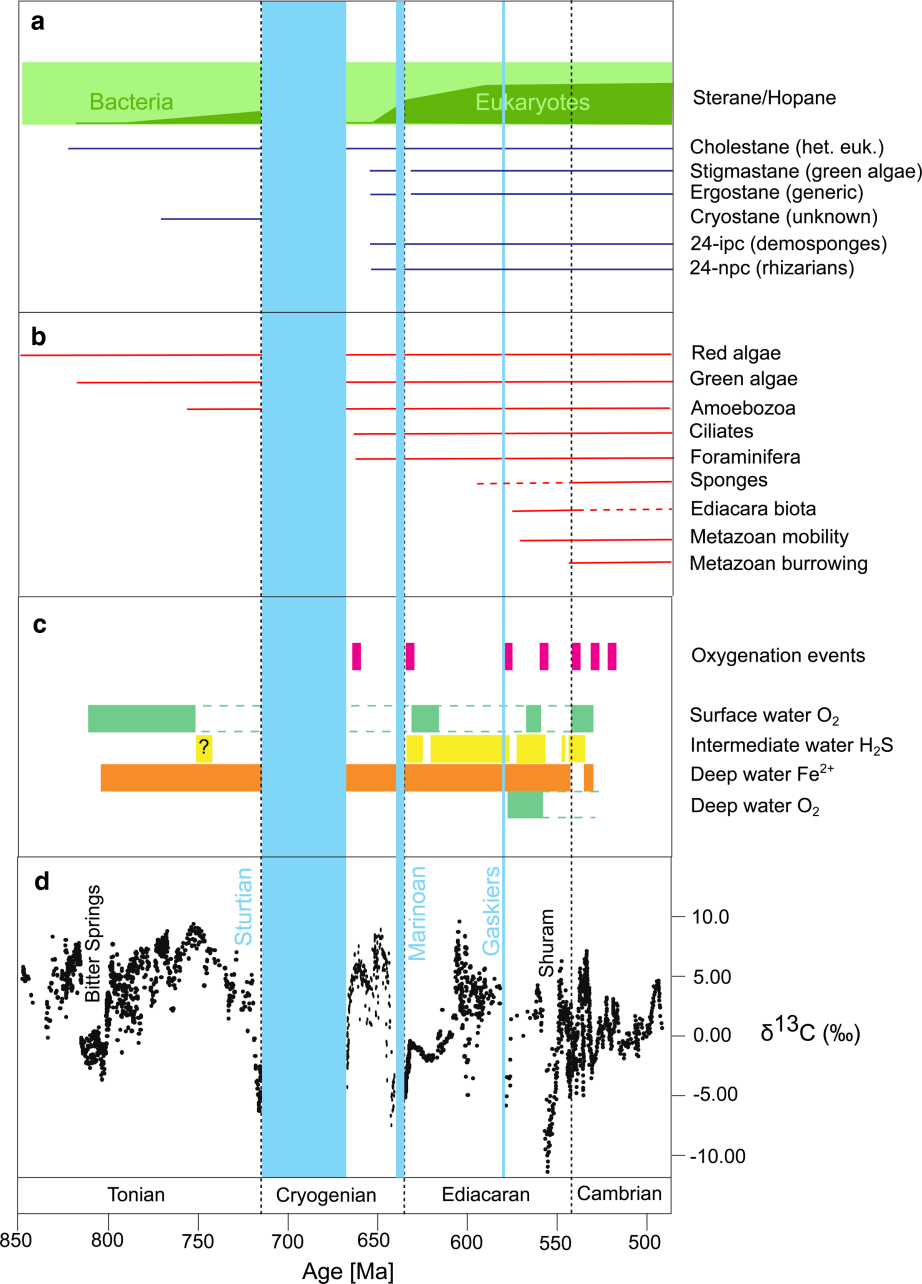
Timeline of biological and environmental changes through the Tonian, Cryogenian, Ediacaran and Cambrian periods (850–500 Ma). (a) Biomarker evidence [34]; sterane/hopane ratio indicates balance of eukaryotes/bacteria, followed by occurrence of eukaryotic steranes (with probable sources [34,35]) including cholestane (heterotrophic eukaryotes), stigmastane (chlorophytes), ergostane (general), cryostane (unknown), 24-ipc = 24-isopropylcholestane (demosponges), 24-npc = 24-*n*-propylcholestane (rhizarians). (b) Fossil evidence, updated from [1] based on studies cited in main text and [33]. (c) Ocean redox state from redox-sensitive elements (pink; global signature) [11,13] and iron speciation data at different depths (green, yellow, orange; local signature) [13,109,110]. (d) The carbon isotopic composition of marine carbonates.

Marine eukaryotes are important ecosystem engineers and their evolution over ∼850–500 Ma (Figure 1a,b) surely had an impact on carbon, phosphorus and oxygen cycling [1]. While a monotonic rise in atmospheric oxygen (pO_2_) in a ‘Neoproterozoic oxygenation event’ [2–5] is still regularly invoked to explain evidence of deeper ocean oxygenation in the Neoproterozoic Era, ocean oxygenation could equally have been caused by declining oxygen demand in deeper waters [1,6]. Here, ‘deeper’ could mean anything below the well-mixed surface layer of stratified shelf seas or the open ocean, but sampling of deep time is largely restricted to continental shelf-slope ocean margins (so does not extend to the deep open ocean) [6]. In deeper waters, oxygenation state is governed by the balance of oxygen supply and respiratory demand from organic matter input. Declining oxygen demand could occur due to: (i) a redistribution of organic matter (and its remineralisation) away from these depths, and/or (ii) a global decline in ocean nutrient levels and organic matter primary production. We focus on phosphorus as the ultimate limiting nutrient, because the ocean available nitrogen inventory — whether as $NO_{3}^{\;-}$, $NH_{4}^{\;+}$ or a mix of both [6] — tends to track changes in the phosphorus inventory [7], even though models predict nitrogen would have been much further below the ‘Redfield ratio’ to phosphorus (required by organisms) in a more anoxic ocean [6,8,9].

Available data show that ocean redox conditions were temporally (as well as spatially) variable in the late Neoproterozoic-early Paleozoic (Figure 1c), while the carbon cycle underwent major fluctuations (Figure 1d). A series of transient ocean oxygenation events are inferred, beginning in the middle of the Cryogenian period (720–635 Ma) [10,11], and getting more frequent through the Ediacaran (635–540 Ma) [12] and early-mid Cambrian (540–500 Ma) [13]. If these transient events were due purely to changes in atmospheric pO_2_, then it must have peaked and declined repeatedly. They could equally have been caused by rapid reorganizations of nutrient and carbon cycling within the ocean, potentially between alternative stable states (anoxic high P recycling, oxic efficient P removal) [14]. Indeed, the onset of oxygenation appears sufficiently rapid in some cases [12] that ∼10^4^ year declines in ocean nutrient inventories look a more plausible explanation than ∼10^6^ year increases in atmospheric pO_2_. These are not mutually exclusive scenarios as rapid biogeochemical reorganisations would ultimately be countered by a slower adjustment of pO_2_ [14].

Here, we explore how eukaryote evolution could have changed the nutrient and redox state of their (submarine) world. We start by introducing key controls on ocean redox state, marine phosphorus cycling and oxygen cycling. Then, we consider possible causes of the rise to ecological prominence of eukaryotic algae, their biogeochemical effects, and those of early sessile animals, and later mobile burrowing animals.

**Figure 2.**
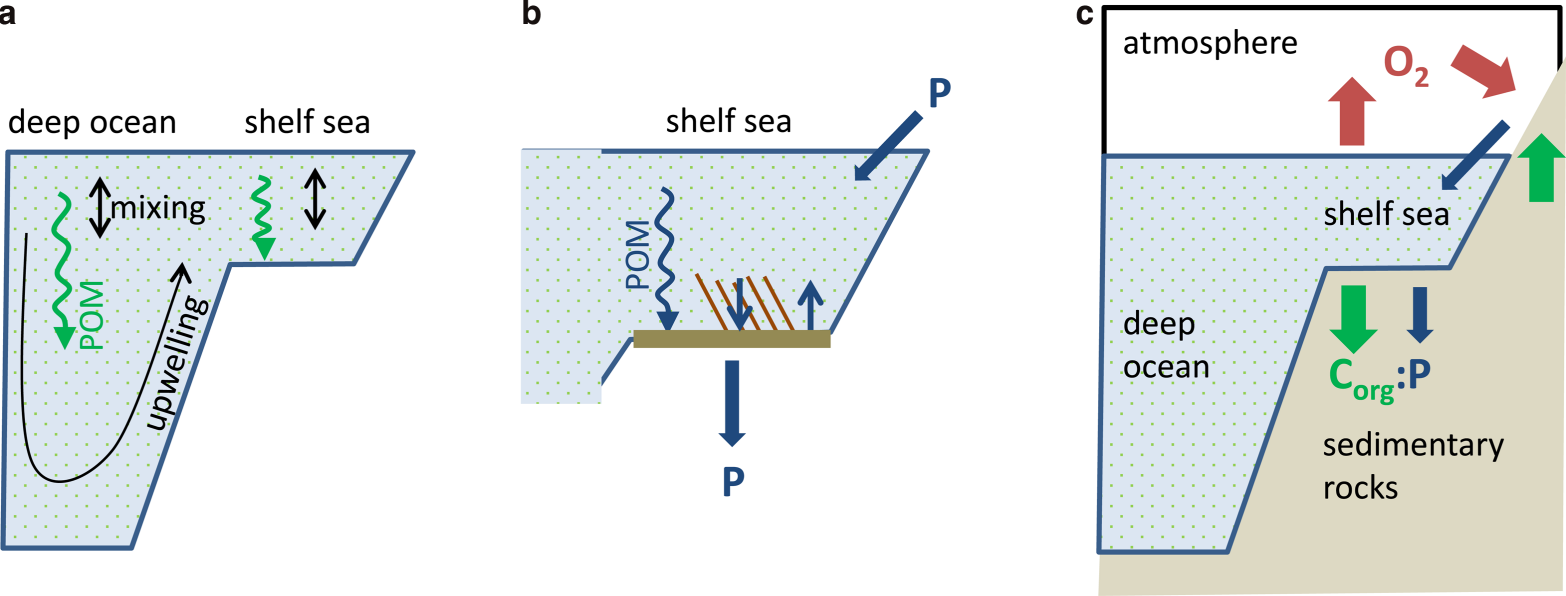
Timescales and processes of eukaryotic effects on biogeochemical cycling. (**a**) Ocean circulation (∼10^3^ year) timescale (black arrows): circulation redistributes O_2_ supply from atmosphere and O_2_ demand from DOM (green dotted background) and sinking POM (green downward wiggle arrow) within the water column. (**b**) Phosphorus cycling (∼10^4^ year) timescale (dark blue arrows), inset of shelf sea: the ocean P inventory adjusts to maintain balance between P input (via weathering) and P burial (primarily on shelves). P sequestration in sediments is enhanced by biological pump (downward wiggle arrow) and by sessile benthic animals (downward arrow). P is preferentially recycled from sediments especially under euxinic conditions (upward arrow). (**c**) Oxygen cycling (∼10^6^ year) timescale (red arrows): Organic carbon burial (*C*_org_; green downward arrow) provides O_2_ source, governed by P input and *C*_org_:P burial ratio which is redox-sensitive. Oxidative weathering of ancient *C*_org_ in sedimentary rocks (green upward arrow) provides O_2_ sink.

## Processes and timescales

We distinguish three timescales over which eukaryotic evolutionary or ecologically driven changes are coupled to biogeochemical cycles (Figure 2):

(1) A ∼10^3^ year ocean circulation timescale [15] of effects on the nature and distribution of organic matter and corresponding oxygen demand in the water column (and between it and the sediments; Figure 2a).
(2) A ∼10^4^ year timescale [16] of changes in P burial efficiency altering the ocean P inventory, which in turn controls the amount of organic matter produced and corresponding oxygen demand (Figure 2b). This modern P timescale could have differed in the Neoproterozoic.
(3) A ∼10^6^ year timescale of changes in the oxygen source from organic carbon (*C*_org_) burial altering atmospheric pO_2_ (Figure 2c), which in turn controls oxygen supply to the water column. This timescale is estimated for the Neoproterozoic based on pO_2_ ∼0.1 PAL (present atmospheric level) supported by ∼2.5 × 10^12^ mol year^−1^ net O_2_ source [17].

When looking at the low temporal resolution of the geological record, we expect that the effects of eukaryote evolution on organic matter cycling (1) cannot be resolved from their effects on phosphorus cycling (2), whereas the slower timescale of changes in atmospheric pO_2_ (3) is resolvable from the others.

Today, a relatively efficient ‘biological pump’ of sinking particulate organic matter (POM) creates oxygen demand across a range of water depths, and transfers *C*_org_ and P to sediments.

On geologic timescales >10^4^ years (i.e. longer than the residence time of P in the ocean), global phosphorus output to sediments must equal global input from rivers (ultimately derived from weathering). This means that changes in P input or the efficiency of P removal (the focus here) cause the ocean P inventory and corresponding output flux to adjust until output again matches input. Today, despite an efficient biological pump, ∼70% of global phosphorus removal occurs in shelf sea settings and only ∼30% on the deep ocean floor [16,18]. Hence, here we ignore P burial in deep ocean sediments and focus on shelf seas, because P burial there largely controls the global P reservoir.

The burial of photosynthetically derived *C*_org_ also occurs mostly in shelf seas [19] and represents the major net source of oxygen to the atmosphere: CO_2_+H_2_O→CH_2_O(↓buried)+O_2_(↑). The *C*_org_ burial flux (*F*_Corg_) is controlled by the phosphorus input/burial flux (*F*_P_; which limits production) and the burial ratio of *C*_org_ to total phosphorus in marine sediments [(*C*_org_/*P*)_burial_; which is redox-sensitive]: *F*_P_ × (*C*_org_/*P*)_burial_ = *F*_Corg_. To first order, we do not expect evolutionary or ecologically driven changes in organic matter cycling within the ocean to have impacted atmospheric pO_2_, because the ocean P inventory adjusts to changes in P burial efficiency such that the P burial flux again matches the (unchanged) P input flux — and the corresponding oxygen source from *C*_org_ burial is left unchanged.

In reality, the ratio of *C*_org_ to total phosphorus buried in new sedimentary rocks, (*C*_org_/*P*)_burial_, can change (thus affecting atmospheric pO_2_), because it is sensitive to the redox state of ocean bottom waters and hence to ocean nutrient levels and (ultimately) atmospheric pO_2_. Under anoxic and euxinic (sulfate-reducing) bottom-water conditions, phosphorus recycling to the water column is enhanced, increasing (*C*_org_/*P*)_burial_ [20,21], and oxygenation reduces P recycling from sediments [22] decreasing (*C*_org_/*P*)_burial_. This acts as a positive feedback amplifying changes in phosphorus levels, productivity and redox state on ∼10^4^ year timescales [23]. However, on ∼10^6^ year timescales, changes in atmospheric pO_2_ provide negative feedback [14,24]. Less clear is what happens to phosphorus cycling under widespread anoxic but ‘ferruginous’ (iron-reducing) conditions [25]. While the formation of Fe(III) oxides and associated trapping of P in sediments will be suppressed, there is evidence that vivianite and mixed Fe(II)–Fe(III) minerals (‘green rust’) can provide a potentially large P sink [26,27]. This could change the sign of the feedback, such that ferruginous conditions enhance P removal suppressing their own spread, but amplify declining pO_2_ [28,29].

## Dissolved organic matter world

**Figure 3.**
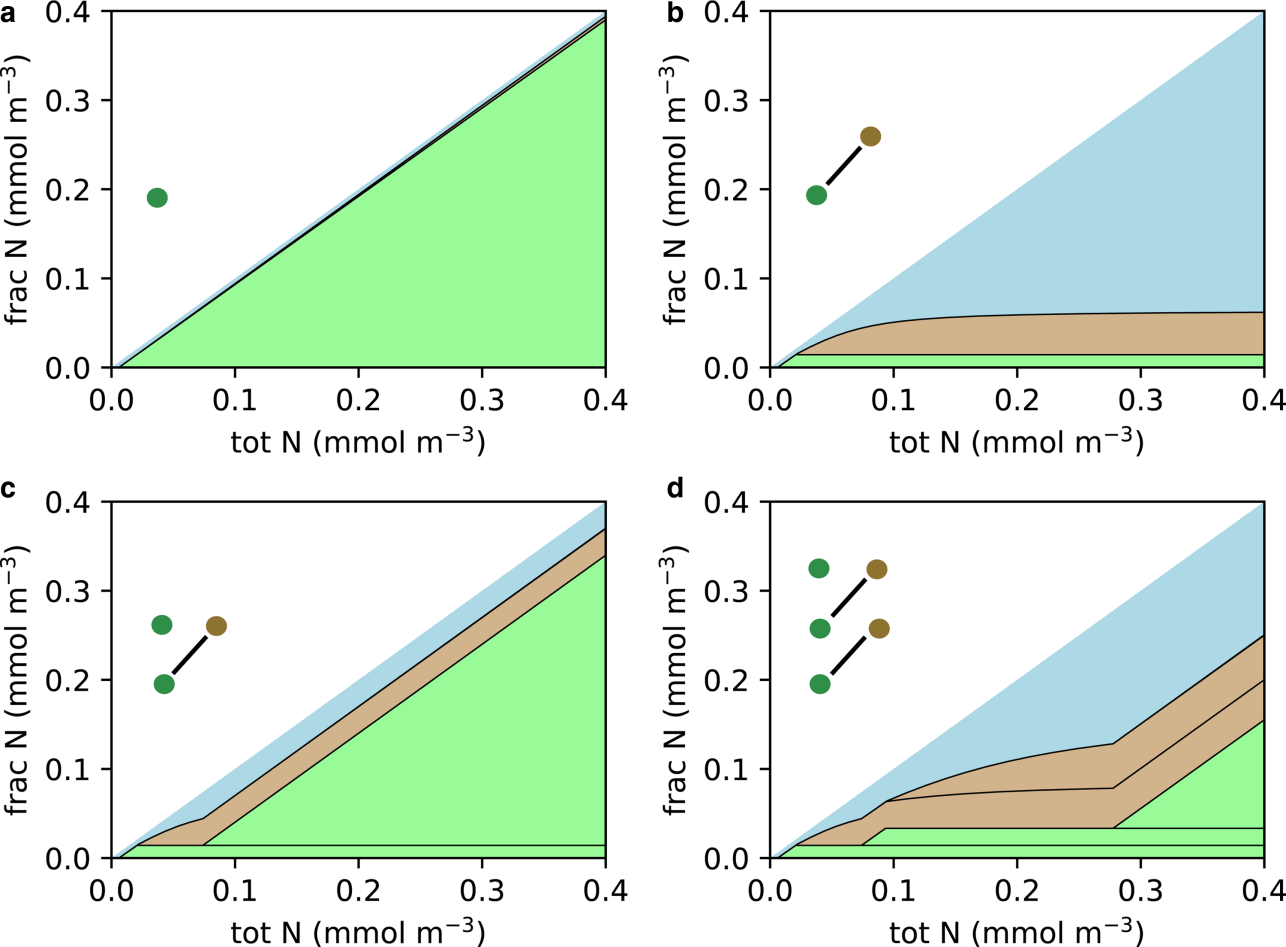
Community size structure in an idealized steady-state microbial food chain model [52,111], for assumed evolutionary steps. Green = autotrophs, brown = protist heterotrophs, blue = dissolved nitrogen. Insets show assumed community composition (dots) in size classes (increasing in size upwards), with lines indicating trophic relationships. Graphs show the fraction of total nitrogen (‘frac N’) in dissolved form (blue) and in each component of the size structured population, as total nitrogen (‘tot N’) increases. Heterophs are stacked on top of autotrophs and for each, where different size classes coexist, the smallest size class is at the bottom with progressively larger size classes stacked on top: (**a**) cyanobacteria only — nutrients are drawn down to limiting concentration and cyanobacterial population increases (until light limitation, not shown); (**b**) including phagotrophy from heterotrophic nanoflagellates limits cyanobacterial population size, allowing nutrient levels to rise; (**c**) this allows autotrophic nanoflagellates to coexist with cyanobacteria; (**d**) eukaryophagy limits population size of autotrophic nanoflagellates and allows larger size classes of eukaryotic phytoplankton (at high total nitrogen levels).

Picture the ocean in the mid to late Tonian period (∼850–720 Ma). Despite the evolution of algae ∼1.7–1.4 Ga [30], including multi-cellular red algae by ∼1.1 Ga [31], and the radiation of red and green algae into the marine environment [32,33], they left no biomarker record [34] (Figure 1a). The low sterane/hopane ratio of biomarkers [34] indicates that bacteria dominated preserved lipids, with cholestane (probably from heterotrophic eukaryotes) and cryostane (of unknown origin [35]) the only eukaryotic steranes present. This requires that unicellular algae were either an ecologically insignificant contributor to marine productivity [34], or that eukaryotic biomass was efficiently recycled either in the water column [36] or in microbial mats [37]. Efficient water column recycling of primary production by prokaryotes (Figure 3a) and possibly small protists (Figure 3c) would have closed a ‘microbial loop’ ensuring dissolved organic matter (DOM; operationally defined as <0.22 or <0.7 µm) [38,39] dominated the water column. This DOM pool was not a massive, ancient one [40], rather it turned over on <10^4^ year timescales consistent with modern observations [38,39].

**Figure 4.**
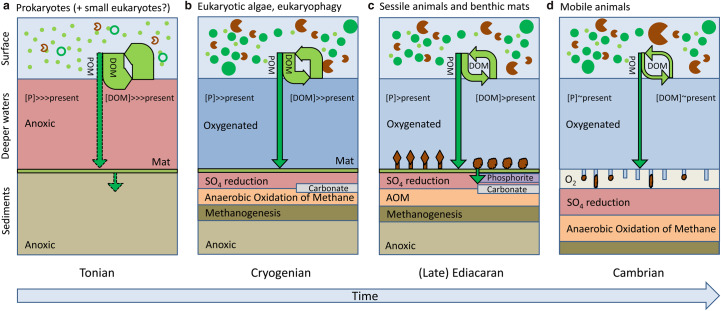
Scenarios for different shelf sea biogeochemical regimes through time, showing redox state after adjustment of P cycle (∼10^4^ year) but before any adjustment of O_2_ cycle. Physical setting is a stratified shelf sea ∼100 m deep with surface waters separated from deeper waters by a sharp thermocline. DOM, dissolved organic matter (pale green). POM, particulate organic matter (dark green). Arrow width roughly represents magnitude of organic matter flux. We assume constant P input flux throughout hence P burial flux into sediments is identical throughout, but this is achieved at different [P] and through a different balance of processes over time. (**a**) Tonian ‘DOM world’ either dominated by cyanobacterial productivity (small pale green dots) or including small green algae (dark green circles) and small phagotrophic eukaryotes (brown ‘Pac-Men’), overlying benthic mats (pale green layer). Dashed arrows indicate uncertainty surrounding POM pathway(s) to sediments from sinking ‘marine snow’ and/or photoautotrophic mats. (**b**) Cryogenian world of eukaryotic algae (larger dark green dots) and eukaryophagy (larger brown ‘Pac-Men’) with a biological pump transferring POM to sediments. (**c**) Late Ediacaran world of sessile animals, including rangeomorph fronds (brown diamonds on stalks) and filter-feeding sponges (brown clouds) transferring POM to sediments. Their location on top of benthic mats creates a sharp redox boundary supporting phosphorite and authigenic carbonate deposition. (**d**) Cambrian world of mobile animals (brown splodges) bioturbating (and thus oxygenating) upper sediments, which lowers the *C*_org_/*P* burial ratio enabling a smaller sedimentary POM flux to maintain the required P output flux.

A microbially dominated DOM recycling ecosystem would have had interesting implications for water column redox state [6]. Unlike the biological pump in the modern ocean (due to sinking POM), oxygen demand in the water column would be strongly influenced by advective and diffusive transport of DOM. This would still be able to drive anoxia in the open-ocean thermocline (see Figure 5f of [6]) and in shelf seas (Figure 4a), although less efficiently for a given nutrient level than particulate export. Shelf-sea environments would be expected to show strong latitudinal patterns in redox state, with permanent anoxia likely in permanently stratified regions at low-mid latitudes, and seasonal anoxia in seasonally stratifying high-latitudes shelves.

With all oxygen demand used up at intermediate depths, deeper waters of the open ocean would be less prone to anoxia (Figure 5f of [6]), having their [O_2_] governed by the balance of supply from the atmosphere via high-latitude deep convection and (a smaller) demand from inorganic reductant input at mid-ocean ridges [6]. Any additional POM flux into deep waters could readily have driven them anoxic (Figure 5d of [6] and [41]). However, what little data we have from the truly deep Proterozoic ocean suggest that some oxygen was present [42,43], consistent with a DOM-dominated system without a significant biological pump [6].

Maintaining an oxygenated Proterozoic atmosphere (given tectonic reductant input) required greater than ∼25% of present-day *C*_org_ burial [17]. A key puzzle is; how did organic matter — including P and C — get out of the bottom of this DOM-cycling system in solid, sedimentary form? Much of it must have been derived from seafloor microbial mats. It seems unlikely that heterotrophic bacterial mats could extract significant DOM from the water column, relative to much larger water column bacterial populations. Instead, occasional aggregation of DOM into POM [44] and of bacterial cells into larger POM, which sunk as ‘marine snow’, seems a more plausible food source for heterotrophic mats. Additionally, autotrophic shallow water mats would have produced POM.

In this ‘DOM world’, balancing the P cycle would have demanded a larger ocean P inventory (Figure 4a) to drive the same P output, consistent with geochemical inferences of high P levels by the Cryogenian [29,45]. As ocean nutrient inventory determines the total amount of organic matter production in the ocean, a high P ocean would have been prone to anoxia at the depths where DOM was respired, particularly under lower early Neoproterozoic atmospheric pO_2_ [6].

## Eukaryotic algae and eukaryophagy

Fossils indicate a diversification of heterotrophic eukaryotes in the late Tonian [46–48], including testate amoebae and organic or siliceous scales, followed by the advent of foraminifera and ciliates in the Cryogenian [49] (Figure 1b). Recent (re)analysis of the biomarker record [34] indicates that steranes from eukaryotic algae, demosponges and rhizarians (a group which includes foraminifera and radiolarians) all first appear in the Cryogenian period (720–635 Ma), between the Sturtian and Marinoan ‘snowball Earth’ glaciations ∼660–640 Ma [50], and the sterane/hopane ratio increases, indicating the prevalence of eukaryotic over bacterial lipids [34] (Figure 1a).

The absence of a eukaryotic algal biomarker record prior to the Cryogenian has been attributed to nutrient scarcity [34,51]. However, in the modern ocean phagotrophy limits cyanobacterial population size allowing eukaryotic algae to coexist [52], where even in the most oligotrophic regions mixotrophic eukaryotes that consume bacteria compete effectively with prokaryotes [53]. Also, during Phanerozoic ocean anoxic events that deplete nitrogen, eukaryotes comprise a significant fraction of productivity [54]. Furthermore, any boost in phosphorus levels during [45] or after the Sturtian glaciation would be transient [1] hence cannot explain the irreversible biomarker transition.

Instead, we suggest two scenarios linking the biomarker record of eukaryotic algae [34] with an apparent escalation in protistan predation [55,56] (Figure 3). In the first scenario, eukaryotic bacterivory was, for some reason, ineffective prior to the Cryogenian (despite being plesiomorphic among eukaryotes), allowing prokaryotic autotrophs to competitively exclude larger eukaryotic algae, because smaller cells are more efficient at diffusion-limited nutrient uptake [52] (Figure 3a). Then, an increase in the effectiveness of eukaryotic bacteriophagy limited cyanobacterial population size (Figure 3b, as in the modern ocean), allowing surface nutrient levels to rise and creating a niche for autotrophic eukaryotes (Figure 3c), followed by a rapid escalation of eukaryophagy to exploit this new resource [57] (Figure 3d). Alternatively, the advent of eukaryophagy put a selection pressure for predation resistance on an earlier cryptic population of small heterotrophic, autotrophic and mixotrophic eukaryotes (Figure 3c), driving increases in size (Figure 3d), armour, and therefore sinking speed and preservation potential — leading to the formation of the algal biomarker record. By filtering out smaller cyanobacterial cells, the advent of sponges [58–60] could also have provided a strong selection pressure for larger eukaryotic algal cells in shelf seas [1].

Thanks to their larger size and faster sinking rate, eukaryotes created a biological pump of POM [1]. The short-term effect would have been to increase oxygen demand in the deeper waters of shelf seas and the open ocean [6,61]. However, by creating an efficient particulate P removal flux to shelf sediments, it would have lowered ocean P content and corresponding O_2_ demand over ∼10^4^ years, thus tending to oxygenate deeper waters of shelf seas (Figure 4b) and the open ocean [6]. This prediction is supported by redox proxy evidence of ocean oxygenation after the Sturtian [10,11,62]. The contribution of redox-sensitive P cycling feedback to this oxygenation is uncertain, but the partial oxygenation of ‘ferruginous’ background ocean waters (Figure 1c) apparently did not cause a major drop in the efficiency of P removal with Fe minerals [27–29], as that would have rapidly counteracted further oxygenation. The oxygenation event is inferred to have reversed before the Marinoan [11], perhaps because partial shelf seafloor oxygenation produced a lower *C*_org_ to total phosphorus burial ratio [21], thus triggering a decline in atmospheric pO_2_.

## Sessile animals and benthic mats

The nature and timing (Figure 1a,b) of the first animals remains contested. Recent molecular phylogenetic studies support the common sense view that sponges (Porifera) are the sister group to all other animals [63,64]. Relaxed molecular clocks put the origin of crown-group demosponges 872–657 Ma (across studies) [65] consistent with biomarkers ∼660–640 Ma [58–60], but put silicification and spicule production later at 648–616 Ma [66]. Hence, the gap to the first widely accepted fossil sponges in the early Cambrian ∼535 Ma [67–69] could be partly due to poor preservation potential. One ∼600 Ma fossil [70] could also close this gap, if supported. A counter-view takes the fossil record at face value (rejecting the molecular clock and biomarker evidence) and argues sponges are not the basal animals and originated in the latest Ediacaran–Cambrian [68,69].

The first complex macroscopic body fossils of the Ediacara biota ∼570–540 Ma are interpreted as a mix of stem- and crown-group animals [71,72]. Early ‘rangeomorph’ fronds that stuck up from the deep, dark seafloor could have fed by osmotrophy [73] — enhanced in deep, low-flow regimes by the establishment of a ‘canopy flow’ regime [74] — facilitating uptake of dissolved nutrients. However, if we accept that the Ediacara biota were eukaryotic, then they would have had other feeding modes — phagocytosis — and their own source of motility — flagella — with which to create advection that can significantly enhance nutrient uptake [75]. Either way, fronds could have provided a spatially concentrated source of POM to sediments (e.g. on death), thus lowering DOM and P concentration in the water column [76].

Whenever actively water-pumping, filter-feeding sponges appeared, they would have altered the size structure of the water column ecosystem, ocean nutrient levels and redox state (Figure 4c). As well as efficiently filtering bacteria and POM, in modern (oligotrophic) coral reef settings, the ‘sponge loop’ [77] converts DOM to POM — forming an effective nutrient concentration and recycling system and a mechanism for transferring POM to sediments [78]. Sponge symbionts also sequester polyphosphate, which can trigger apatite formation, thus removing P to sediments [79]. Thus, when sponges arose they could have provided a significant direct pathway of C and P to sediments, lowering overall oxygen demand in the water column and thus tending to oxygenate it. *Thectardis* ∼565–555 Ma has been interpreted as a possible sponge [80], although others disagree [68,69]. Subsequently, there is geochemical evidence for the progressive expansion of siliceous sponges on the Yangtze Platform ∼550–525 Ma, and associated decrease in the DOC pool, enhanced *C*_org_ and phosphorus burial, and water column oxygenation [81].

The combination of an oxygenated shelf water column overlying a sediment surface still covered by mats (and sessile animals) produced a shelf-sea ecosystem structure unique to the Neoproterozoic, with a sharp sediment-surface redox gradient that would favour the formation of authigenic carbonate [82] and phosphorite [83–85]. In modern environments, sulphide-oxidising bacterial mats (*Thiomargarita*, *Beggiatoa*) that bridge the water-sediment interface accumulate polyphosphate from oxic waters and utilise it under anoxic conditions, triggering apatite precipitation [86,87]. Neoproterozoic phosphorites are typically associated with stromatolites [88,89] and some contain filamentous microfossils that resemble modern sulphide-oxidising bacteria [90]. Phosphorites are associated with nearshore oxygen oases ∼610 Ma [91], then shift to greater depths and areal extent ∼570 Ma onwards (e.g. the Doushantuo Formation) [83,91].

## Mobile animals

The first mobile trace-makers that scratched across mat surfaces appeared ∼565 Ma [92], but did not significantly disrupt the ‘mat seal’ on the sediments, until the first burrowing animals evolved. Fine meiofaunal traces from 555 to 542 Ma have recently been described [93], and widespread burrows capable of significant sediment mixing slightly predate the Precambrian/Cambrian boundary [94].

Bioturbating animals are well-known ‘ecosystem engineers’ [95] that bring oxygen into contact with sediments, increase the turnover rate of *C*_org_ and O_2_ [96], suppressing sulfate reduction [97] and trapping phosphorus in iron oxides [98], while also increasing water exchange fluxes that release nitrogen and phosphorus to the water column [97]. It is hypothesised that by oxidising upper sediment layers, the evolution of bioturbators increased the sulfate inventory of the ocean [99], and by lowering (*C*_org_/*P*)_burial_, initially removed phosphorus and oxygenated the ocean (∼10^4^ years) (Figure 4d) and then lowered atmospheric pO_2_ and deoxygenated the ocean (∼10^7^ years) restoring higher P levels [100]. What is unresolved is when these predicted effects became globally significant.

One view is that bioturbation increased in depth and intensity in the Cambrian ‘agronomic revolution’ in Stages 2–4 (∼530–510 Ma) [101,102], causing initial ocean oxygenation and then declining atmospheric pO_2_ and ocean deoxygenation over the next ∼20 Myr [100]. A counter view is that the development of bioturbation only became globally significant from the late Silurian ∼420 Ma onwards [103,104]. The argument depends on whether sediment P cycling responds linearly or non-linearly to increasing burrowing depth. Diagenetic modelling suggests the effects of burrowing animals are nonlinear and even shallow bioturbation significantly sequesters P [105].

These alternative hypotheses make distinct, testable predictions. Evidence that mid-depth waters of the Yangtze Platform oxygenated from Cambrian stages 2–4 has been used to question the early bioturbation model [106], but is actually consistent with the original predictions [100], which show that ocean oxygenation should accompany the initial onset of deep bioturbation (Stages 2–4), followed by a much slower deoxygenation (governed by the slow timescale of atmospheric pO_2_ decline). Wider evidence shows an ocean oxygenation event ∼520 Ma (broadly coincident with the ‘Cambrian explosion’) followed by deoxygenation [13,107,108].

The Cambrian evolution of large zooplankton would also have increased the efficiency of the biological pump [36], transferring organic matter to sediments, lowering the ocean P inventory and tending to oxygenate the ocean [1,107].

## Conclusion

The Neoproterozoic–Cambrian transition was not unidirectional or driven solely by either rising atmospheric pO_2_ or evolutionary innovations. We describe a series of eukaryotic innovations that created and strengthened the biological pump of POM from the ocean to the sediments, with major consequences for the phosphorus, carbon and oxygen cycles. We suggest that each phase of eukaryote evolution tended to lower P levels and oxygenate the ocean on ∼10^4^ year timescales, but by decreasing *C*_org_/*P* burial ratios tended to lower atmospheric pO_2_ and deoxygenate the ocean again on ∼10^6^ year timescales. Coupled with tectonic drivers, and biogeochemical and ecological feedback, potentially between alternate stable states [14], this could help explain the transient nature and ∼10^6^ year duration of oceanic oxygenation events through the Cryogenian–Ediacaran–Cambrian.

## Summary

- The late Tonian ocean ∼750 Ma was dominated by rapid microbial cycling of DOM with elevated nutrient (P) levels due to inefficient organic matter removal to sediments.
- We hypothesise that abrupt onset of the eukaryotic algal biomarker record in the Cryogenian ∼660–640 Ma was linked to an escalation of protozoan predation (eukaryophagy).
- This ecological regime shift created a ‘biological pump’ of sinking POM, which transferred *C*_org_ and P to sediments.
- The Late Ediacaran advent of sessile benthic animals on top of microbial mats increased the efficiency of *C*_org_ and P transfer to sediments, contributing to the deposition of phosphorites.
- The Cambrian explosion of mobile burrowing animals broke the ‘mat seal’ on the upper sediments but by oxygenating them it enabled alternative means of efficient P retention.
- Each phase of eukaryote evolution tended to lower P levels and oxygenate the ocean on ∼10^4^ year timescales, but by decreasing *C*_org_/P burial ratios, tended to lower atmospheric pO_2_ and deoxygenate the ocean again on ∼10^6^ year timescales.
- This can help explain the transient nature and ∼10^6^ year duration of oceanic oxygenation events through the Cryogenian–Ediacaran–Cambrian periods.

## Abbreviations

*C*_org_: organic carbon
DOM: dissolved organic matter
POM: particulate organic matter
